# 5′,6-Dichloro-1′,3′,3′-trimethyl­spiro­[2*H*-1-benzopyran-2,2′-indoline]

**DOI:** 10.1107/S1600536808018722

**Published:** 2008-07-09

**Authors:** Nameer Alhashimy, Helge Müller-Bunz, Benjamin Schazmann, Dermot Diamond

**Affiliations:** a Adaptive Sensors Group, National Centre of Sensor Research, School of Chemical Sciences, Dublin City University, Dublin 9, Ireland; b School of Chemistry and Chemical Biology, University College Dublin, Belfield, Dublin 4, Ireland

## Abstract

In the crystal structure of the title compound, C_19_H_17_Cl_2_NO, the indoline and benzopyran ring systems are approximately perpendicular to each other. The indoline ring is in an envelope conformation with the spiro C atom as the flap. The N atom of the indoline ring forms a pyramidal environment, the sum of the angles at this atom being 352.46°.

## Related literature

For related literature, see: Crano & Guglielmetti (1999 ▶); Kholmanskii & Dyumanev (1987 ▶); Tamai & Miyasaka (2000 ▶); Krongauz *et al.* (2000 ▶); Minkin (2004 ▶); Crano *et al.* (1996 ▶); Dvornikov *et al.* (1994 ▶); Tamai & Miyasaka (2000 ▶); Yoshida & Morinaka (1994 ▶); Willner *et al.* (1993 ▶); Byrne *et al.* (2006*a* ▶,*b* ▶); Raić-Malić *et al.* (2004 ▶); Aldoshin & Atovmyan (1985 ▶); Aldoshin *et al.* (1987 ▶); Mannschreeck *et al.* (1999 ▶). For the synthesis of the title compound, see: Martin *et al.* (1998 ▶).
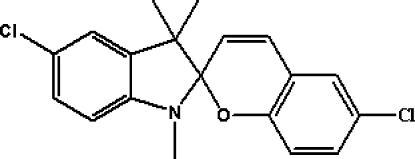


## Experimental

### 

#### Crystal data

C_19_H_17_Cl_2_NO
*M* *_r_* = 346.24
Monoclinic, 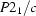

*a* = 8.3105 (7) Å
*b* = 18.2576 (16) Å
*c* = 11.1921 (10) Å
β = 104.770 (2)°
*V* = 1642.1 (2) Å^3^
*Z* = 4
Mo *K*α radiation
μ = 0.40 mm^−1^
*T* = 100 (2) K
0.50 × 0.40 × 0.05 mm

#### Data collection

Bruker SMART CCD area-detector diffractometer
Absorption correction: multi-scan (**SADABS**; Sheldrick, 2000 ▶) *T*_min_ = 0.740, *T*_max_ = 0.980
16175 measured reflections
4312 independent reflections
3857 reflections with *I* > 2σ(*I*)
*R*_int_ = 0.023

#### Refinement

*R*[*F* ^2^ > 2σ(*F* ^2^)] = 0.046
*wR*(*F* ^2^) = 0.122
*S* = 1.05
4312 reflections
276 parameters
All H-atom parameters refined
Δρ_max_ = 0.96 e Å^−3^
Δρ_min_ = −0.25 e Å^−3^

### 

Data collection: *SMART* (Bruker, 2007 ▶); cell refinement: *SAINT* (Bruker, 2007 ▶); data reduction: *SAINT*; program(s) used to solve structure: *SHELXS97* (Sheldrick, 2008 ▶); program(s) used to refine structure: *SHELXL97* (Sheldrick, 2008 ▶); molecular graphics: *DIAMOND* (Brandenburg, 2001 ▶); software used to prepare material for publication: *publCIF* (Westrip, 2008 ▶).

## Supplementary Material

Crystal structure: contains datablocks I, global. DOI: 10.1107/S1600536808018722/nc2106sup1.cif

Structure factors: contains datablocks I. DOI: 10.1107/S1600536808018722/nc2106Isup2.hkl

Additional supplementary materials:  crystallographic information; 3D view; checkCIF report

## Figures and Tables

**Table 1:** Selected interplanar angles (°) for the title compound

Atoms defining plane 1	Atoms defining plane 2	Interplanar angle
C2, C6, C8, N	C11, C19, O	85.03 (4)
C3, C4, C8, N	C8, C11, N	28.9 (1)
C1, C2, C3, C4, C5, C6	C3, C4, C8, N	2.4 (1)

**Fig. 1.**
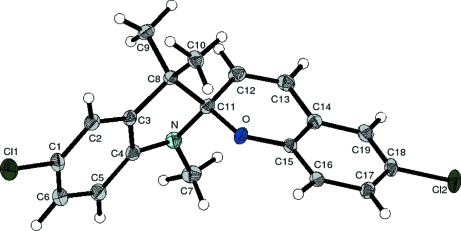
A perspective view of the asymmetric unit of title compound, showing the atom numbering and thermal ellipsoids at a 50% probability level.

## supplementary crystallographic information

### Comment

Spiropyrans are a family of organic photochromic compounds (Carno & Guglielmetti, 1998). This family of compounds are well studied and documented (Kholmanskii & Dyumanev, 1987; Tamai & Miyasaka, 2000; Krongauz *et al.*, 2000; Minkin, 2004) because they can be converted from a closed colourless form into a strongly coloured open form using UV irradiation. This tremendous characteristic of spiropyran compounds has been utilized by scientists for many applications such as light-sensitive eyewear (Crano *et al.*, 1996), high density optical storage (Dvornikov *et al.*,1994), molecular switches (Tamai & Miyasaka, 2000, Minkin, 2004) and molecular devices (Yoshida & Morinaka, 1994; Willner *et al.*, 1993). Our main interest was utilizing spiropyran derivatives as transducers in optical sensors, where selective binding to certain metal ions was achieved. The binding and release of such ions can be controlled by exposure to light of around 380 nm (open form) and 550 nm (close form) respectively (Byrne *et al.*, 2006*a*; Byrne *et al.*, 2006*b*). The title compound was envisaged as an intermediate in the synthesis of further spiropyran derivatives, whereby the chlorides groups can be replaced by substitution with variety of functional groups. The title compound consists of two molecular fragments: An indoline ring linked to a benzopyran ring by the spiro (C11) atom (Fig 1). The two fragments are almost perpendicular to each other (Table 2). The bond lengths of (C11—N) and (C11—O) are both approximately equal, which agrees with previous reports (Raić-Malić *et al.*, 2004; Aldoshin & Atovmyan, 1985; Aldoshin *et al.*, 1987). The spiro carbon atom (C11) is out of the plane of the other four indoline ring atoms (Table 2). The indoline ring is quite coplanar with the fused benzene ring (Table 2). The sum of the angles of the nitrogen atom at the indoline moiety is 352.46°, which indicates a pyramidal arrangement about this atom. These results are in agreement with previous reports (Raić-Malić *et al.*, 2004).

### Experimental

The title compound was originally synthesized according to a method outlined in a patent (Martin *et al.*, 1998). Our procedure differs from the original synthesis, especially with regard to the purification process. Single crystals suitable for X-ray diffraction were grown by slow evaporation from ethanol solution.

To 5-chlorosalicylaldehyde (1.53 g, 9.6 mmol) in 10 ml e thanol, a solution of 5-chloro-2-methylene-1,3,3-trimethylindoline (1.95 ml, 9.6 mmol) in 20 ml of ethanol was added slowly, over 30 min. This reaction mixture was heated to reflux over 24 h and then cooled down to ambient temperature. The solvent was evaporated by vacuum and the resulting crude compound was purified by column chromatography from the system solvent of 1:5, ethyl acetate: hexane, yielding a white powder (2.30 g, 69.4%).

### Refinement

All hydrogen atoms were located in the difference fourier map and allowed to refine isotropic without any restraints. C—H bond lenghts vary from 0.92 (2) to 1.00 (2) Å.

### Crystal data

**Table:**

C_19_H_17_Cl_2_NO	*F*_000_ = 720
*M**_r_* = 346.24	*D*_x_ = 1.401 Mg m^−^^3^
Monoclinic, *P*2_1_/*c*	Mo *K*α radiation λ = 0.71073 Å
Hall symbol: -P 2ybc	Cell parameters from 7373 reflections
*a* = 8.3105 (7) Å	θ = 2.2–31.7º
*b* = 18.2576 (16) Å	µ = 0.40 mm^−^^1^
*c* = 11.1921 (10) Å	*T* = 100 (2) K
β = 104.770 (2)º	Plate, colourless
*V* = 1642.1 (2) Å^3^	0.50 × 0.40 × 0.05 mm
*Z* = 4	

### Data collection

**Table:**

Bruker SMART CCD area-detector diffractometer	4312 independent reflections
Radiation source: fine-focus sealed tube	3857 reflections with *I* > 2σ(*I*)
Monochromator: graphite	*R*_int_ = 0.023
Detector resolution: 8.366 pixels mm^-1^	θ_max_ = 29.0º
*T* = 100(2) K	θ_min_ = 2.2º
φ and ω scans	*h* = −11→11
Absorption correction: multi-scan(*SADABS*; Sheldrick, 2000)	*k* = −24→24
*T*_min_ = 0.740, *T*_max_ = 0.980	*l* = −15→15
16175 measured reflections	

### Refinement

**Table:**

Refinement on *F*^2^	Secondary atom site location: difference Fourier map
Least-squares matrix: full	Hydrogen site location: difference Fourier map
*R*[*F*^2^ > 2σ(*F*^2^)] = 0.046	All H-atom parameters refined
*wR*(*F*^2^) = 0.122	*w* = 1/[σ^2^(*F*_o_^2^) + (0.0653*P*)^2^ + 1.2622*P*] where *P* = (*F*_o_^2^ + 2*F*_c_^2^)/3
*S* = 1.05	(Δ/σ)_max_ = 0.002
4312 reflections	Δρ_max_ = 0.96 e Å^−^^3^
276 parameters	Δρ_min_ = −0.25 e Å^−^^3^
Primary atom site location: structure-invariant direct methods	Extinction correction: none

### Special details

**Table:**

Experimental. ^1^H NMR δ(CDCl_3_); 1.190 (S, 3H, CH_3_), 1.30 (S, 3H, CH~3~),2.72 (S, 3H, CH_3_), 5.73 (d, 1H, *J*= 10.4 Hz, CH=CH), 6.46 ( d, H, *J*= 12.8 Hz, Ar-H), 6.67 (d, 1H, *J *=9.6 Hz, Ar-H), 6.83 (d, 1H, *J *= 16.4, Ar-H), 7.01-7.08 ( m, 2H, Ar-H), 7.15 ( d, 1H, *J *= 10.4 Hz, CH=CH).^13^C NMR δ(CDCl_3_); 19.95, 25.68, 29.04, 51.96, 104.60, 107.78, 116.33, 119.91,120.17, 122.11, 123.93, 124.85, 126.28, 127.37, 128.80, 129.50, 138.53, 146.73,152.80.M.S. (m/z ion) (m/z 346.2).
Geometry. All e.s.d.'s (except the e.s.d. in the dihedral angle between two l.s. planes) are estimated using the full covariance matrix. The cell e.s.d.'s are taken into account individually in the estimation of e.s.d.'s in distances, angles and torsion angles; correlations between e.s.d.'s in cell parameters are only used when they are defined by crystal symmetry. An approximate (isotropic) treatment of cell e.s.d.'s is used for estimating e.s.d.'s involving l.s. planes.
Refinement. Refinement of *F*^2^ against ALL reflections. The weighted *R*-factor *wR* and goodness of fit *S* are based on *F*^2^, conventional *R*-factors *R* are based on *F*, with *F* set to zero for negative *F*^2^. The threshold expression of *F*^2^ > σ(*F*^2^) is used only for calculating *R*-factors(gt) *etc*. and is not relevant to the choice of reflections for refinement. *R*-factors based on *F*^2^ are statistically about twice as large as those based on *F*, and *R*- factors based on ALL data will be even larger.

### Fractional atomic coordinates and isotropic or equivalent isotropic displacement parameters (Å^2^)

**Table:**

	*x*	*y*	*z*	*U*_iso_*/*U*_eq_	
Cl1	1.29108 (6)	0.46363 (2)	0.01666 (4)	0.02892 (13)	
C1	1.2126 (2)	0.38771 (9)	0.07835 (15)	0.0198 (3)	
C2	1.0760 (2)	0.39650 (9)	0.12870 (15)	0.0187 (3)	
H2	1.029 (3)	0.4437 (13)	0.133 (2)	0.025 (5)*	
C3	1.01780 (19)	0.33524 (8)	0.17644 (14)	0.0160 (3)	
C4	1.09359 (19)	0.26690 (8)	0.17419 (14)	0.0169 (3)	
C5	1.2295 (2)	0.25851 (9)	0.12441 (16)	0.0202 (3)	
H5	1.284 (3)	0.2115 (12)	0.120 (2)	0.020 (5)*	
C6	1.2886 (2)	0.32043 (10)	0.07558 (16)	0.0223 (3)	
H6	1.381 (3)	0.3155 (12)	0.037 (2)	0.024 (5)*	
N	1.01339 (17)	0.21423 (7)	0.22743 (13)	0.0186 (3)	
C7	1.0459 (2)	0.13674 (9)	0.21878 (17)	0.0225 (3)	
H7A	1.015 (3)	0.1214 (13)	0.136 (2)	0.029 (6)*	
H7B	0.989 (3)	0.1089 (13)	0.271 (2)	0.030 (6)*	
H7C	1.163 (3)	0.1284 (14)	0.256 (2)	0.038 (7)*	
C8	0.88187 (19)	0.32673 (8)	0.24368 (14)	0.0150 (3)	
C9	0.9546 (2)	0.34836 (9)	0.37930 (15)	0.0197 (3)	
H9A	0.989 (3)	0.3981 (13)	0.383 (2)	0.024 (5)*	
H9B	0.870 (3)	0.3426 (12)	0.427 (2)	0.027 (6)*	
H9C	1.051 (3)	0.3191 (13)	0.419 (2)	0.029 (6)*	
C10	0.7265 (2)	0.37244 (10)	0.18951 (16)	0.0204 (3)	
H10A	0.687 (3)	0.3635 (12)	0.100 (2)	0.022 (5)*	
H10B	0.756 (3)	0.4243 (13)	0.202 (2)	0.028 (6)*	
H10C	0.647 (3)	0.3607 (12)	0.233 (2)	0.023 (5)*	
C11	0.85232 (19)	0.24213 (9)	0.23280 (14)	0.0163 (3)	
O	0.73274 (14)	0.23176 (6)	0.11246 (10)	0.0178 (2)	
C12	0.7959 (2)	0.20568 (9)	0.33518 (15)	0.0194 (3)	
H12	0.853 (3)	0.2181 (12)	0.417 (2)	0.025 (5)*	
C13	0.6788 (2)	0.15405 (9)	0.31410 (15)	0.0200 (3)	
H13	0.651 (3)	0.1291 (13)	0.381 (2)	0.028 (6)*	
C14	0.5891 (2)	0.13478 (8)	0.18904 (15)	0.0166 (3)	
C15	0.61892 (19)	0.17667 (8)	0.09238 (14)	0.0147 (3)	
C16	0.52518 (19)	0.16618 (9)	−0.02828 (14)	0.0159 (3)	
H16	0.550 (3)	0.1972 (12)	−0.090 (2)	0.021 (5)*	
C17	0.4051 (2)	0.11154 (9)	−0.05435 (15)	0.0185 (3)	
H17	0.345 (2)	0.1032 (11)	−0.1356 (18)	0.014 (5)*	
C18	0.38016 (19)	0.06811 (8)	0.04128 (16)	0.0183 (3)	
Cl2	0.23079 (5)	−0.00073 (2)	0.00933 (4)	0.02523 (13)	
C19	0.4695 (2)	0.07902 (9)	0.16192 (16)	0.0188 (3)	
H19	0.452 (3)	0.0512 (12)	0.226 (2)	0.022 (5)*	

### Atomic displacement parameters (Å^2^)

**Table:**

	*U*^11^	*U*^22^	*U*^33^	*U*^12^	*U*^13^	*U*^23^
Cl1	0.0321 (2)	0.0223 (2)	0.0349 (2)	−0.00767 (16)	0.01332 (18)	0.00340 (17)
C1	0.0173 (7)	0.0211 (8)	0.0209 (7)	−0.0046 (6)	0.0047 (6)	0.0022 (6)
C2	0.0180 (7)	0.0160 (7)	0.0208 (7)	−0.0002 (6)	0.0026 (6)	−0.0016 (6)
C3	0.0129 (7)	0.0172 (7)	0.0165 (7)	−0.0009 (5)	0.0015 (5)	−0.0014 (5)
C4	0.0145 (7)	0.0176 (7)	0.0171 (7)	−0.0003 (5)	0.0016 (5)	−0.0016 (5)
C5	0.0152 (7)	0.0200 (8)	0.0248 (8)	0.0016 (6)	0.0041 (6)	−0.0025 (6)
C6	0.0174 (8)	0.0249 (8)	0.0248 (8)	−0.0022 (6)	0.0061 (6)	−0.0025 (6)
N	0.0187 (6)	0.0149 (6)	0.0224 (7)	0.0011 (5)	0.0054 (5)	−0.0002 (5)
C7	0.0284 (9)	0.0143 (7)	0.0252 (8)	0.0031 (6)	0.0078 (7)	0.0011 (6)
C8	0.0141 (7)	0.0149 (7)	0.0158 (7)	−0.0007 (5)	0.0033 (5)	−0.0021 (5)
C9	0.0205 (8)	0.0199 (8)	0.0178 (7)	−0.0011 (6)	0.0032 (6)	−0.0037 (6)
C10	0.0157 (7)	0.0213 (8)	0.0240 (8)	0.0019 (6)	0.0048 (6)	0.0012 (6)
C11	0.0179 (7)	0.0166 (7)	0.0134 (7)	−0.0020 (5)	0.0022 (5)	−0.0011 (5)
O	0.0204 (5)	0.0194 (6)	0.0125 (5)	−0.0082 (4)	0.0023 (4)	0.0001 (4)
C12	0.0235 (8)	0.0199 (7)	0.0145 (7)	−0.0006 (6)	0.0045 (6)	−0.0002 (6)
C13	0.0231 (8)	0.0203 (8)	0.0180 (7)	−0.0001 (6)	0.0080 (6)	0.0026 (6)
C14	0.0169 (7)	0.0147 (7)	0.0190 (7)	0.0010 (5)	0.0058 (6)	0.0003 (5)
C15	0.0133 (6)	0.0134 (6)	0.0182 (7)	−0.0001 (5)	0.0056 (5)	−0.0013 (5)
C16	0.0132 (7)	0.0173 (7)	0.0171 (7)	0.0004 (5)	0.0039 (5)	−0.0006 (5)
C17	0.0143 (7)	0.0182 (7)	0.0216 (8)	0.0001 (6)	0.0018 (6)	−0.0028 (6)
C18	0.0122 (7)	0.0117 (6)	0.0308 (8)	−0.0002 (5)	0.0052 (6)	−0.0012 (6)
Cl2	0.0158 (2)	0.01421 (19)	0.0436 (3)	−0.00319 (13)	0.00372 (17)	0.00058 (15)
C19	0.0179 (7)	0.0146 (7)	0.0256 (8)	0.0007 (6)	0.0086 (6)	0.0034 (6)

### Geometric parameters (Å, °)

**Table:**

Cl1—C1	1.7474 (17)	C9—H9C	0.97 (2)
C1—C6	1.385 (2)	C10—H10A	0.98 (2)
C1—C2	1.399 (2)	C10—H10B	0.98 (2)
C2—C3	1.379 (2)	C10—H10C	0.94 (2)
C2—H2	0.95 (2)	C11—O	1.4680 (18)
C3—C4	1.401 (2)	C11—C12	1.500 (2)
C3—C8	1.517 (2)	O—C15	1.3596 (18)
C4—N	1.388 (2)	C12—C13	1.332 (2)
C4—C5	1.389 (2)	C12—H12	0.94 (2)
C5—C6	1.398 (2)	C13—C14	1.451 (2)
C5—H5	0.98 (2)	C13—H13	0.95 (2)
C6—H6	0.98 (2)	C14—C15	1.397 (2)
N—C11	1.448 (2)	C14—C19	1.401 (2)
N—C7	1.448 (2)	C15—C16	1.390 (2)
C7—H7A	0.94 (2)	C16—C17	1.389 (2)
C7—H7B	0.98 (2)	C16—H16	0.96 (2)
C7—H7C	0.96 (3)	C17—C18	1.389 (2)
C8—C10	1.527 (2)	C17—H17	0.93 (2)
C8—C9	1.535 (2)	C18—C19	1.379 (2)
C8—C11	1.564 (2)	C18—Cl2	1.7384 (16)
C9—H9A	0.95 (2)	C19—H19	0.92 (2)
C9—H9B	1.00 (2)		
			
C6—C1—C2	122.16 (15)	H9B—C9—H9C	107.9 (19)
C6—C1—Cl1	118.44 (13)	C8—C10—H10A	109.9 (13)
C2—C1—Cl1	119.41 (13)	C8—C10—H10B	108.3 (14)
C3—C2—C1	117.62 (15)	H10A—C10—H10B	108.3 (18)
C3—C2—H2	121.6 (14)	C8—C10—H10C	107.7 (14)
C1—C2—H2	120.7 (14)	H10A—C10—H10C	113.3 (18)
C2—C3—C4	120.77 (15)	H10B—C10—H10C	109.2 (19)
C2—C3—C8	130.88 (14)	N—C11—O	109.47 (12)
C4—C3—C8	108.24 (13)	N—C11—C12	110.36 (13)
N—C4—C5	128.63 (15)	O—C11—C12	111.93 (13)
N—C4—C3	110.00 (14)	N—C11—C8	102.84 (12)
C5—C4—C3	121.37 (15)	O—C11—C8	104.77 (12)
C4—C5—C6	118.05 (15)	C12—C11—C8	116.89 (13)
C4—C5—H5	123.3 (13)	C15—O—C11	121.79 (12)
C6—C5—H5	118.6 (13)	C13—C12—C11	122.36 (15)
C1—C6—C5	120.02 (15)	C13—C12—H12	120.5 (14)
C1—C6—H6	120.4 (13)	C11—C12—H12	117.0 (14)
C5—C6—H6	119.5 (13)	C12—C13—C14	120.98 (15)
C4—N—C11	108.93 (13)	C12—C13—H13	120.8 (14)
C4—N—C7	122.00 (14)	C14—C13—H13	118.2 (14)
C11—N—C7	122.46 (14)	C15—C14—C19	119.07 (15)
N—C7—H7A	110.4 (14)	C15—C14—C13	117.73 (14)
N—C7—H7B	109.9 (14)	C19—C14—C13	123.13 (15)
H7A—C7—H7B	112 (2)	O—C15—C16	117.17 (14)
N—C7—H7C	108.0 (16)	O—C15—C14	122.00 (14)
H7A—C7—H7C	112 (2)	C16—C15—C14	120.72 (14)
H7B—C7—H7C	104 (2)	C17—C16—C15	119.88 (15)
C3—C8—C10	114.10 (13)	C17—C16—H16	123.2 (13)
C3—C8—C9	107.98 (13)	C15—C16—H16	116.9 (13)
C10—C8—C9	109.39 (13)	C16—C17—C18	119.23 (15)
C3—C8—C11	100.63 (12)	C16—C17—H17	119.9 (12)
C10—C8—C11	114.13 (13)	C18—C17—H17	120.8 (12)
C9—C8—C11	110.21 (13)	C19—C18—C17	121.53 (15)
C8—C9—H9A	109.3 (13)	C19—C18—Cl2	118.93 (13)
C8—C9—H9B	110.6 (13)	C17—C18—Cl2	119.53 (13)
H9A—C9—H9B	108.9 (19)	C18—C19—C14	119.48 (15)
C8—C9—H9C	112.7 (14)	C18—C19—H19	121.8 (14)
H9A—C9—H9C	107.3 (19)	C14—C19—H19	118.7 (14)
			
C6—C1—C2—C3	0.0 (2)	C9—C8—C11—N	−84.95 (15)
Cl1—C1—C2—C3	179.99 (12)	C3—C8—C11—O	−85.57 (13)
C1—C2—C3—C4	0.0 (2)	C10—C8—C11—O	37.07 (17)
C1—C2—C3—C8	175.66 (15)	C9—C8—C11—O	160.62 (12)
C2—C3—C4—N	179.88 (14)	C3—C8—C11—C12	149.91 (14)
C8—C3—C4—N	3.35 (17)	C10—C8—C11—C12	−87.45 (17)
C2—C3—C4—C5	0.2 (2)	C9—C8—C11—C12	36.10 (19)
C8—C3—C4—C5	−176.36 (14)	N—C11—O—C15	102.17 (16)
N—C4—C5—C6	179.99 (16)	C12—C11—O—C15	−20.53 (19)
C3—C4—C5—C6	−0.4 (2)	C8—C11—O—C15	−148.14 (13)
C2—C1—C6—C5	−0.2 (3)	N—C11—C12—C13	−104.55 (18)
Cl1—C1—C6—C5	179.82 (13)	O—C11—C12—C13	17.6 (2)
C4—C5—C6—C1	0.4 (3)	C8—C11—C12—C13	138.45 (16)
C5—C4—N—C11	−163.27 (16)	C11—C12—C13—C14	−5.4 (3)
C3—C4—N—C11	17.04 (17)	C12—C13—C14—C15	−5.7 (2)
C5—C4—N—C7	−11.2 (3)	C12—C13—C14—C19	177.48 (16)
C3—C4—N—C7	169.10 (15)	C11—O—C15—C16	−172.39 (13)
C2—C3—C8—C10	41.2 (2)	C11—O—C15—C14	11.3 (2)
C4—C3—C8—C10	−142.78 (14)	C19—C14—C15—O	179.72 (14)
C2—C3—C8—C9	−80.7 (2)	C13—C14—C15—O	2.8 (2)
C4—C3—C8—C9	95.37 (15)	C19—C14—C15—C16	3.6 (2)
C2—C3—C8—C11	163.81 (16)	C13—C14—C15—C16	−173.34 (14)
C4—C3—C8—C11	−20.12 (15)	O—C15—C16—C17	−178.97 (14)
C4—N—C11—O	81.89 (15)	C14—C15—C16—C17	−2.7 (2)
C7—N—C11—O	−70.01 (18)	C15—C16—C17—C18	0.2 (2)
C4—N—C11—C12	−154.49 (13)	C16—C17—C18—C19	1.4 (2)
C7—N—C11—C12	53.62 (19)	C16—C17—C18—Cl2	−179.66 (12)
C4—N—C11—C8	−29.08 (16)	C17—C18—C19—C14	−0.4 (2)
C7—N—C11—C8	179.02 (14)	Cl2—C18—C19—C14	−179.39 (12)
C3—C8—C11—N	28.87 (14)	C15—C14—C19—C18	−2.0 (2)
C10—C8—C11—N	151.51 (13)	C13—C14—C19—C18	174.72 (15)

### Table 2 Selected interplanar angles for the title compound

**Table:**

Atoms defining plane 1	Atoms defining plane 2	Interplanar angle (°)
C2, C6, C8, N	C11, C19, O	85.03 (4)
C3, C4, C8, N	C8, C11, N	28.9 (1)
C1, C2, C3, C4, C5, C6	C3, C4, C8, N	2.4 (1)
